# A Comparative Study of Cerium(III) and Cerium(IV) Phosphates for Sunscreens

**DOI:** 10.3390/molecules29092157

**Published:** 2024-05-06

**Authors:** Taisiya O. Kozlova, Darya N. Vasilyeva, Daniil A. Kozlov, Irina V. Kolesnik, Maria A. Teplonogova, Ilya V. Tronev, Ekaterina D. Sheichenko, Maria R. Protsenko, Danil D. Kolmanovich, Olga S. Ivanova, Alexander E. Baranchikov, Vladimir K. Ivanov

**Affiliations:** 1 Kurnakov Institute of General and Inorganic Chemistry of the Russian Academy of Sciences, Moscow 119991, Russia; 2 Faculty of Chemistry, National Research University Higher School of Economics, Moscow 101000, Russia; 3 Faculty of Materials Science, Lomonosov Moscow State University, Moscow 119991, Russia; 4 Institute of Theoretical and Experimental Biophysics of the Russian Academy of Sciences, Pushchino 142290, Russia; 5 Frumkin Institute of Physical Chemistry and Electrochemistry of the Russian Academy of Sciences, Moscow 119071, Russia

**Keywords:** cerium, UV shielding, photocatalytic activity, biocompatibility

## Abstract

Crystalline cerium(III) phosphate (CePO_4_), cerium(IV) phosphates, and nanocrystalline ceria are considered to be promising components of sunscreen cosmetics. This paper reports on a study in which, for the first time, a quantitative comparative analysis was performed of the UV-shielding properties of CePO_4_, Ce(PO_4_)(HPO_4_)_0.5_(H_2_O)_0.5_, and CePO_4_/CeO_2_ composites. Both the sun protection factor and protection factor against UV-A radiation of the materials were determined. Ce(PO_4_)(HPO_4_)_0.5_(H_2_O)_0.5_ was shown to have a sun protection factor of 2.9, which is comparable with that of nanocrystalline ceria and three times higher than the sun protection factor of CePO_4_. Composites containing both cerium dioxide and CePO_4_ demonstrated higher sun protection factors (up to 1.8) than individual CePO_4_. When compared with the TiO_2_ Aeroxide P25 reference sample, cerium(III) and cerium(IV) phosphates demonstrated negligible photocatalytic activity. A cytotoxicity analysis performed using two mammalian cell lines, hMSc and NCTC L929, showed that CePO_4_, Ce(PO_4_)(HPO_4_)_0.5_(H_2_O)_0.5_, and nanocrystalline ceria were all non-toxic. The results of this comparative study indicate that cerium(IV) phosphate Ce(PO_4_)(HPO_4_)_0.5_(H_2_O)_0.5_ is more advantageous for use in sunscreens than either cerium(III) phosphate or CePO_4_/CeO_2_ composites, due to its improved UV-shielding properties and low photocatalytic activity.

## 1. Introduction

Excessive exposure to sunlight, especially to its ultraviolet component (200–400 nm), has a harmful effect on skin and can lead to premature ageing, erythema, sunburn, and even cancer [1]. To prevent skin damage, it is recommended to use sunscreens containing UV filters—organic or inorganic substances capable of reducing the negative effects of sunlight, due to a combination of various chemical or physical factors [2]. Organic UV filters include various compounds, e.g., cinnamates, avobenzone, octocrylene, salicylates, benzophenones, etc. Despite their diverse chemical composition, organic UV filters share several characteristics that may pose a risk to human health. These include a tendency toward photodegradation under UV light, a proclivity to penetrate the skin barrier and exert a systemic effect on the body, as well as the potential to cause allergic reactions, endocrine disorders, and other issues [3,4]. In contrast, inorganic UV filters possess a higher photostability than organic compounds, lack the ability to penetrate the skin, and are the most recommended in terms of safety for sunscreen applications. Today, the most widely used inorganic UV filters are titanium dioxide and zinc oxide, which provide protection against solar radiation in a wide spectral range, especially in comparison with organic filters [5]. However, as numerous studies have shown, under certain conditions, these oxides themselves negatively affect the skin, for example, due to the photocatalytic generation of reactive oxygen species, which impart multiple adverse effects [6]. Recently, nanocrystalline cerium dioxide, ceria-based solid solutions, and calcium or cerium phosphates [7,8,9,10] have been proposed as safer alternatives to TiO_2_ and ZnO materials. Notably, phosphate materials barely interact with organic phosphate-rich compounds that are present in sunscreen formulations, which improves stability and extends the shelf life of the latter [11].

In previous studies regarding cerium phosphates as UV-protective materials, much attention was paid to Ce(III) compounds [12,13,14,15,16], and only a few reports were devoted to Ce(IV) phosphates [17,18] or cerium phosphate composites with CeO_2_ [19,20]. All of these materials have been shown to possess a high-enough absorption in the UV range and extremely low photocatalytic activity, which makes them suitable for use in sunscreen cosmetics. 

In the present study, to evaluate the performance of cerium-based components of sunscreens, a comparative analysis was conducted of the UV-filter properties of cerium(IV) phosphate, Ce(PO_4_)(HPO_4_)_0.5_(H_2_O)_0.5_; cerium(III) phosphate, CePO_4_ (monazite); CePO_4_/CeO_2_ composites; and nanocrystalline CeO_2_ (reference sample). The sun protection factor (SPF) and protection factor against UV-A radiation (UVAPF) were selected as quantitative measures, to enable the findings to be comparable with similar studies. This analysis was supplemented by an evaluation of the photocatalytic activity of these materials and their cytotoxicity, to determine the most preferable cerium-based compound for use in sunscreens.

## 2. Experimental Section

The following materials were used, as received, without further purification: Ce(NO_3_)_3_·6H_2_O (pure grade, Lanhit, Moscow, Russia), phosphoric acid (85 wt.% aq, *ρ* = 1.689 g/cm^3^, extra-pure grade, Komponent-Reaktiv, Moscow, Russia), sodium hydroxide (high purity grade, Sigma-Aldrich, St. Louis, MO, USA), aqueous ammonia (25 wt.% aq, extra-pure grade, Khimmed, Moscow, Russia), isopropanol (extra-pure grade, Khimmed), and distilled water.

**Figure 1:**
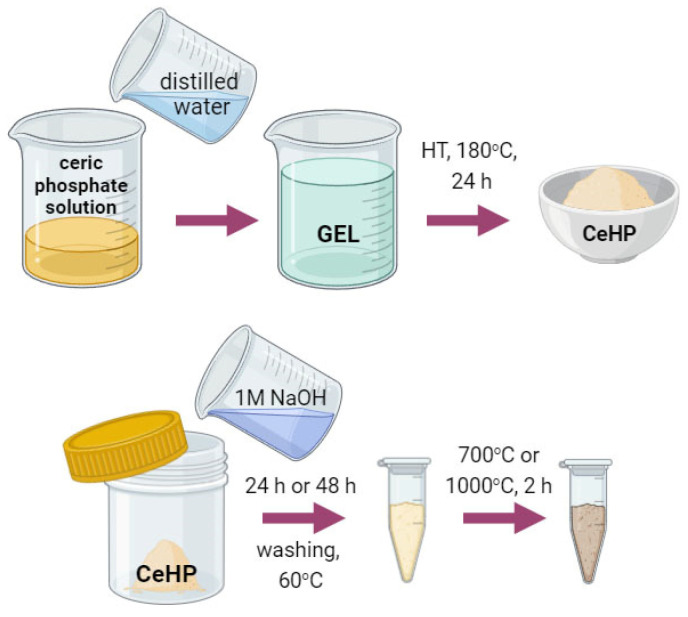
The scheme of the synthesis of ceric phosphate, Ce(PO_4_)(HPO_4_)_0.5_(H_2_O)_0.5_, and CePO_4_/CeO_2_ composites.

Nanocrystalline CeO_2_, which was used for the synthesis of crystalline ceric phosphate Ce(PO_4_)(HPO_4_)_0.5_(H_2_O)_0.5_ and as a reference sample, was obtained in accordance with the previously published procedure [21]. Briefly, 0.08 M cerium(III) nitrate solution in aqueous isopropanol (water:isopropanol = 1:1 *v*/*v*) was mixed with 3 M aqueous ammonia. The yellow precipitate obtained was washed with distilled water to a neutral pH and dried at 60 °C. As a second reference sample, CeO_2_ annealed at 700 °C for 2 h was used (labelled as CeO_2_-700). Crystalline Ce(PO_4_)(HPO_4_)_0.5_(H_2_O)_0.5_ (hereinafter referred to as CeHP) was synthesised by hydrothermal treatment of ceric phosphate solution at 180 °C for 24 h [22]. Composites of cerium(III) phosphate and ceria (CePO_4_/CeO_2_) were obtained as reported earlier [22]. Briefly, 1 M NaOH solution (107 mL) was added to 0.16 g of ceric phosphate Ce(PO_4_)(HPO_4_)_0.5_(H_2_O)_0.5_ and allowed to stand in a closed vessel, with stirring, for 48 or 96 h. Each of the samples obtained was washed several times with distilled water and dried at 60 °C for 24 h in air. Dried products were subjected to thermal treatment in a muffle furnace by linear heating (5 °C/min) to 700 °C or 1000 °C and were kept for 2 h in air (see Figure 1, Table 1). A Ce^III^PO_4_ sample (hereinafter referred to as CeP) was produced by thermal treatment of Ce(PO_4_)(HPO_4_)_0.5_(H_2_O)_0.5_ at 1000 °C (heating rate 5 °C/min) for 2 h in air.

Powder X-ray diffraction (PXRD) patterns were acquired with a DX-2700BH (Haoyuan, Dandong, China) diffractometer, using Cu K_α1,2_ radiation in the 2θ range of 5°–80° with 0.02° 2θ steps and a counting time of no less than 0.2 s per step. The identification of the diffraction peaks was carried out using the ICDD database. The full-profile analysis of diffraction patterns, with quantitative determination of the crystal phases ratio and the size of coherent scattering regions, was performed using the Rietveld method and the MAUD software package (version 2.99). 

Scanning electron microscopy (SEM) images were obtained using an Amber GMH (Tescan, Brno, Czech Republic) or an NVision 40 (Carl Zeiss, Oberkochen, Germany) microscope, operated at an accelerating voltage of 1 kV using an Everhart–Thorney secondary electron detector or a backscattered electron InLens detector. Energy-dispersive X-ray spectroscopy (EDX) data were obtained using an Ultim Max (Oxford Instruments, Abingdon, UK) detector at an accelerating voltage of 20 kV.

Fourier-transform infrared (FTIR) spectra of the samples were recorded using an InfraLUM FT-08 (Lumex, St. Petersburg, Russia) spectrometer in the range of 400–4000 cm^−1^ in attenuated total reflectance mode.

Raman spectra were obtained using a Confotech NR-500 spectrometer (SOL Instruments, Minsk, Belarus) with 532 nm laser excitation using 20× objective (NA = 0.45) at ~2 mW laser power. 

Absorption spectra of CeO_2_, CeHP, CeP, and CePO_4_/CeO_2_ composites were registered in a diffuse reflection mode on a Lambda 950 (PerkinElmer, Waltham, MA, USA) spectrometer in the 200–1000 nm range. The SPF and UVAPF values were determined in accordance with the ISO 24443-2016 international standard [23], which implies the possibility of establishing the values of sun protection factors that correlate with in vivo changes, using spectral measurements in vitro and mathematical modelling. The measurement procedure and calculation formulae have been reported elsewhere [18]. Sample preparation for this study included the following steps: 10 wt.% suspensions containing 0.06 g of cerium phosphates, or composites, or CeO_2_ powders and 0.54 g of a solution consisting of 9.9 wt.% H_2_O, 90 wt.% glycerol, and 0.1 wt.% sodium dodecyl sulfate were prepared in an agate mortar and used for further analysis; 0.035 g of each suspension was evenly spread over the surface of the 3M Transpore Tape 1527-2 film (27.5 cm^2^ area). The total transmittance spectra of the films coated with the suspensions were recorded on a Lambda 950 (PerkinElmer, Waltham, MA, USA) spectrometer using an integrating sphere (150 mm diameter) in the range of 290–400 nm; 3M Transpore Tape 1527-2 coated with a similar formulation, with neither cerium phosphates nor other cerium-containing materials, was used as a reference sample. For each material, measurements were repeated at least four times, using similarly prepared tapes, and then averaging the resulting values. A Suntest CPS+ (ATLAS MTS, Linsengericht, Germany) device was used as a source of UV radiation.

The photocatalytic degradation of methylene blue under UV–visible light irradiation was chosen as a model reaction to assess the photocatalytic activity of the samples. An HPX-2000 (OceanOptics, Orlando, FL, USA) xenon lamp was used as the light source. In a typical experiment, 1 mg of the sample was dispersed in 2 mL of deionised water and the mixture was stirred for 30 min (*t* = 37 °C). Then, 0.1 mL of 100 mg/L methylene blue (MB) solution was added and the suspension obtained was stirred for 45 min in the dark. The suspension was subsequently irradiated for 4 h and the concentration of MB was monitored by registering UV–visible spectra every minute, using an QE65000 (OceanOptics, Orlando, FL, USA) spectrometer. The kinetics of methylene blue degradation was described by a first-order equation and photocatalytic activity was determined as the decomposition rate constant normalised to the photocatalyst’s weight. Commercial photocatalyst TiO_2_ Aeroxide P25 (Evonik, Essen, Germany) was used as a reference sample.

Since the cytotoxicity of CeP and nanocrystalline CeO_2_ powders had been recently assessed [18], here, a similar study was performed for the Ce^III^PO_4_ sample using cell cultures of human mesenchymal stem cells and line NCTC L929 mouse fibroblasts. The cells were seeded in 96-well plates at a density of 20,000 cells/cm^2^ in a DMEM/F12 culture medium containing 10% fetal bovine serum. After 12 h of cultivation, the nutrient medium was replaced with an identical medium containing Ce^III^PO_4_ suspended in the medium, using a magnetic stirrer for 30 min. The concentrations of Ce^III^PO_4_ in DMEM/F12 medium were 0.125 mg/mL, 0.25 mg/mL, 0.5 mg/mL, and 1 mg/mL. In a control experiment, the culture medium was replaced with a fresh medium that did not contain cerium(III) phosphate.

The MTT test was used to assess the viability of the cells. After 48 h, the nutrient medium with Ce^III^PO_4_ was replaced with a serum-free culture medium DMEM/F12 containing tetrazolium salt at a concentration of 0.5 mg/mL. After incubation of the plate for 3 h at 37 °C (5% CO_2_), the culture medium was removed and 100 μL of DMSO was added. The plates were shaken, at room temperature, for 10 min (200 rpm) to dissolve the formazan crystals. The optical density of formazan was measured on a BIO-RAD 680 photometer at 540 nm. Statistical data processing was performed using GraphPad Prism 8 software. Statistically significant differences were determined in accordance with the Mann–Whitney U-test.

The differential staining of human mesenchymal stem cells using fluorescent dyes SYTO 9 (green) and propidium iodide (red) was used to assess the proportion of dead cells. During the experiment, the dyes were dissolved in Hanks’ balanced salt solution (HBSS). Cells were stained using a dye concentration of 5 μM. Then, the cells were incubated for 15 min at 37 °C in a humidified atmosphere (5% CO_2_) and images were taken using a Zeiss 200 M inverted fluorescence microscope. The micrographs were further processed using ImageJ software (version 1.53i).

## 3. Results and Discussion

**Figure 2:**
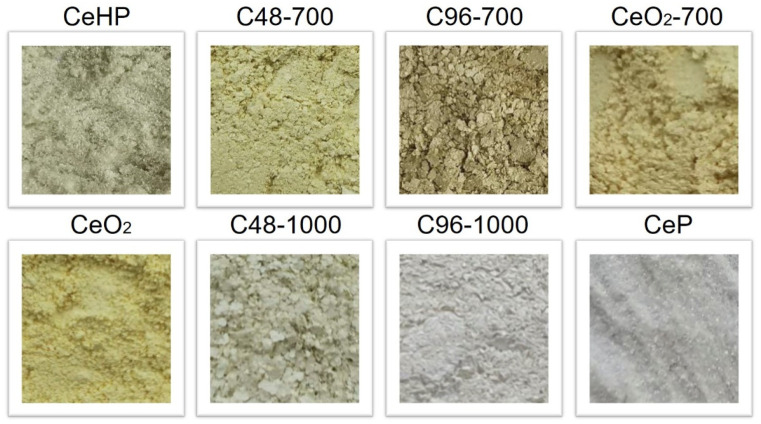
Appearance of ceria, cerium phosphates, and CePO_4_/CeO_2_ composites (optical images).

The powders obtained differed in colour: yellow grey (CeHP), yellow and brown yellow (initial ceria, CeO_2_-700, C48-700, and C96-700 samples), grey beige (C48-1000, and C96-1000 composites) and white grey (CeP), as illustrated in Figure 2. Note that, for cosmetic applications, it is strongly recommended to use UV filters with a natural appearance of skin [24].

**Figure 3:**
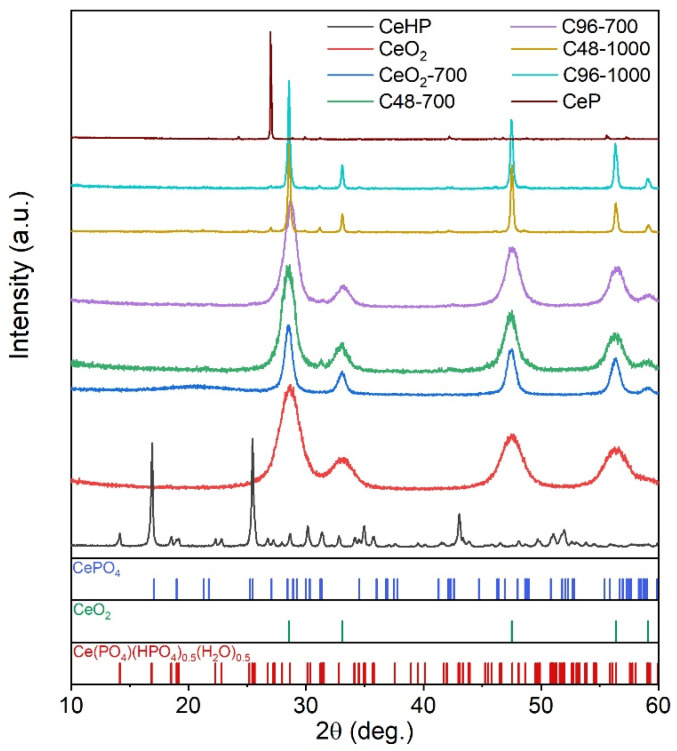
Diffraction patterns of ceria, cerium phosphates, and CePO_4_/CeO_2_ composites.

According to the X-ray diffraction data (Figure 3), the reference ceria samples (CeO_2_, and CeO_2_-700) and CeHP were a single-phase cerium dioxide (ICDD 34-0394) and Ce(PO_4_)(HPO_4_)_0.5_(H_2_O)_0.5_ [25], respectively. The CeP sample was a highly textured CePO_4_ (monazite, ICDD 32-199) with a small (4.1 ± 0.3 wt.%) admixture of CeP_3_O_9_ (ICDD 33-0336). This minor admixture would not significantly affect the absorbance spectrum of the product (CeP_3_O_9_ band gap is nearly 6 eV [26]), so any differences changes in its SPF value compared with pure CePO_4_ are expected to be negligible. Samples C48-700, C48-100 C96-700, and C96-1000 contained a mixture of CeO_2_ and CePO_4_ with various crystal phase ratios (see Table 2). An increase in the annealing temperature from 700 °C to 1000 °C led to a significant increase in ceria particle sizes in composites, from ten nanometres to more than 100 nanometres. At the same time, the duration of CeHP alkaline hydrolysis (48 or 96 h) had virtually no effect on the phase composition and crystallite sizes of the composites.

**Figure 4:**
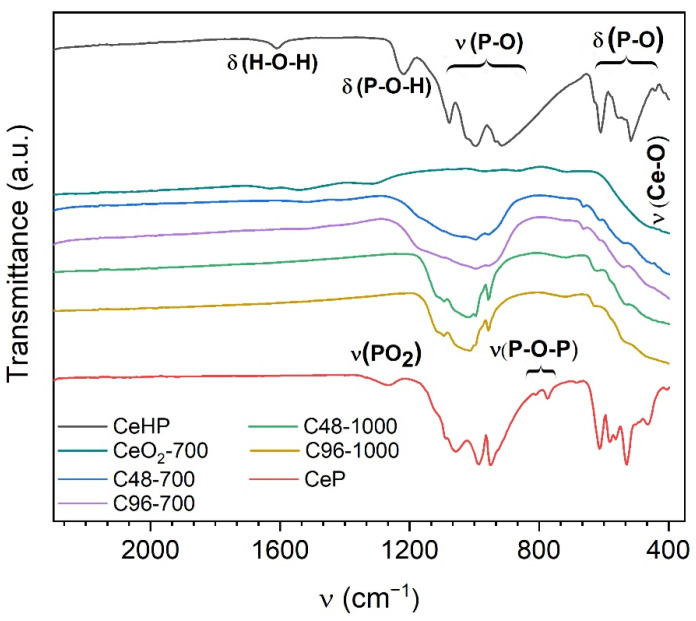
IR spectra of CeO_2_, cerium phosphates, and composite samples.

IR spectra of cerium(III) and cerium(IV) phosphates, as well as CePO_4_/CeO_2_ composites, are presented in Figure 4. The IR spectrum of the CeHP sample coincides well with the Ce(PO_4_)(HPO_4_)_0.5_(H_2_O)_0.5_ spectrum [27,28] and comprises two major absorption bands at 1100–900 cm^−1^ and 650–440 cm^−1^, which correspond to the stretching and deformational vibrations of the phosphate anion, respectively [29]. In the IR spectrum of the CeP sample, in addition to the characteristic absorption bands of the phosphate anions [30], there are two weak absorption bands, at 1270 cm^−1^ and 770 cm^−1^, which can be attributed to metaphosphate group vibrations [31,32]. The presence of characteristic absorption bands at 1100–900 cm^−1^ and 650–440 cm^−1^ in the IR spectrum of the C96-700 composite indicates phosphate anions and indirectly confirms the formation of a CePO_4_ admixture, which was not detected by XRD analysis. 

**Figure 5:**
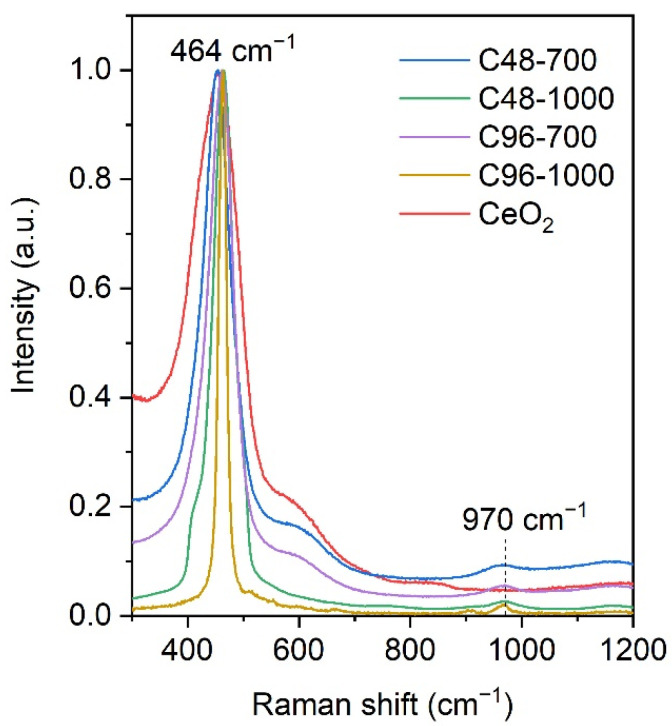
Raman spectra of ceria and composites samples.

The presence of the absorption band at 468 cm^−1^ in the Raman spectra of ceria samples and CePO_4_/CeO_2_ composites (Figure 5) is definitely associated with the F_2g_ band of CeO_2_ [33]. The characteristic peak at 464 cm^−1^ in the spectra of CePO_4_/CeO_2_ composites is narrower and more symmetrical than in the spectrum of pure ceria, while its half-width decreases with increasing annealing temperature. The observed trend was caused by an increase in the size of CeO_2_ crystallites [34], which was confirmed by XRD data. The broad band at 550–600 cm^−1^ observed in the spectra of ceria powder, C48-700, and C96-700 composites could be due to the oxygen defects in CeO_2_ nanocrystals [35]. The peak observed at 970 cm^−1^ confirms the presence of phosphate groups in the composites [29]. 

**Figure 6:**
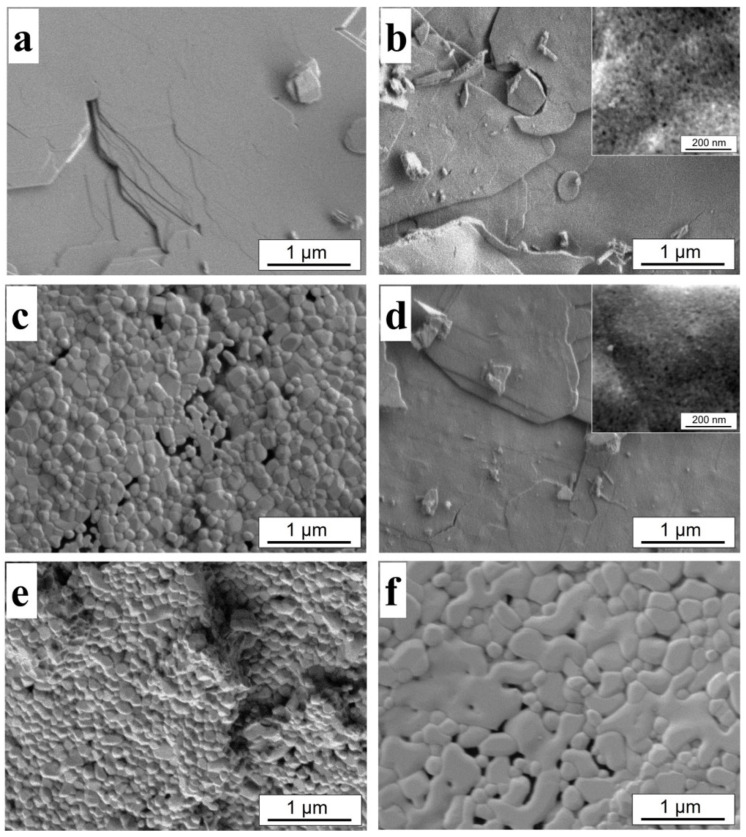
SEM images of (**a**) CeHP, (**b**) C48-700, (**c**) C48-1000, (**d**) C96-700, (**e**) C96-1000, and (**f**) CeP samples.

Scanning electron microscopy data for the CeHP sample and its thermolysis products, including CePO_4_/CeO_2_ composites, are presented in Figure 6. CeHP, C48-700, and C96-700 samples consist of lamellar aggregates. Such a microstructure of inorganic UV filters, according to Sato et al. [36], ensures comfort when applying sun protection products to the skin. Powders obtained at 1000 °C consisted of aggregated grains forming a lamellar motif.

EDX data show that the Ce:P ratio for the CeHP and CeP samples was close to the nominal compositions (1:1.5 and 1:1, respectively). For all the composites, the presence of phosphorus was detected, with the content of cerium being approximately seven times higher than that of phosphorus (Table S1, Supplementary Materials). This additionally confirms the formation of cerium dioxide in the composites.

**Figure 7:**
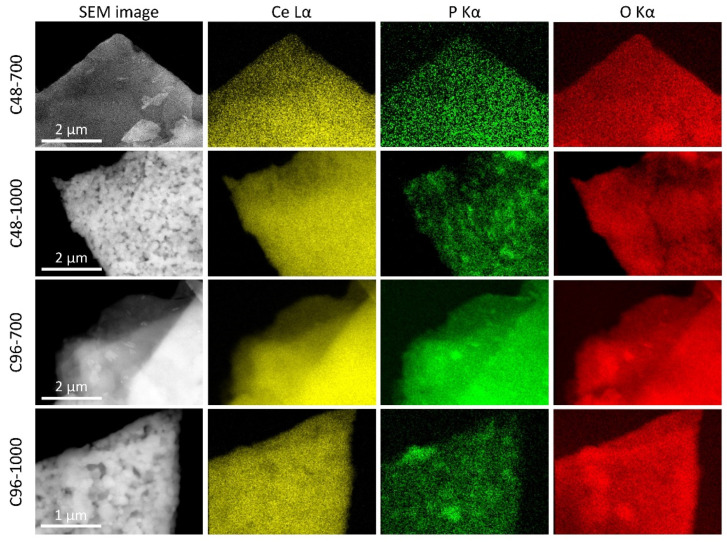
SEM images in the backscattered electron mode for the CePO_4_/CeO_2_ composites and corresponding EDX maps of cerium (yellow), phosphorous (green), and oxygen (red).

The distribution of phases in the obtained composites was studied using SEM-EDX mapping (Figure 7). In the sample heated at 700 °C, cerium, phosphorus, and oxygen are uniformly distributed in the lamellar particles of the composites. For the sample heated at 1000 °C, the SEM images taken at the backscattered electron mode show that, on the surface of the lamellar particles, the bright particles with a higher average atomic number are present, most likely cerium oxide. The dark areas in the corresponding SEM images show a higher phosphorus content (see EDX maps), while cerium and oxygen are uniformly distributed. It can, therefore, be concluded that, on the phosphate surface, the formation of CeO_2_ nanoparticles occurs after etching in aqueous NaOH and subsequent treatment at 700 °C. At 1000 °C, relatively large CeO_2_ particles form on the surface of phosphate-rich lamellar aggregates, which consist of crystalline monazite.

The UV–vis absorption spectra of the powders (Figure S1) show the characteristic wide bands below 400 nm for the ceria and CeHP samples, as well as for the CeP sample, corresponding to charge-transfer transitions between the O(2p) and Ce(4f) states [37,38] or the 4f^1^-5d^1^ electron transition of Ce^+3^ [39,40], respectively. For the CePO_4_/CeO_2_ composites, the absorption spectra virtually coincide with the corresponding spectrum of the CeO_2_ sample, while, in the composites, a slight shift in the absorption band edge can be observed, apparently due to the presence of Ce^+3^ and structural defects [41,42,43,44,45,46].

**Figure 8:**
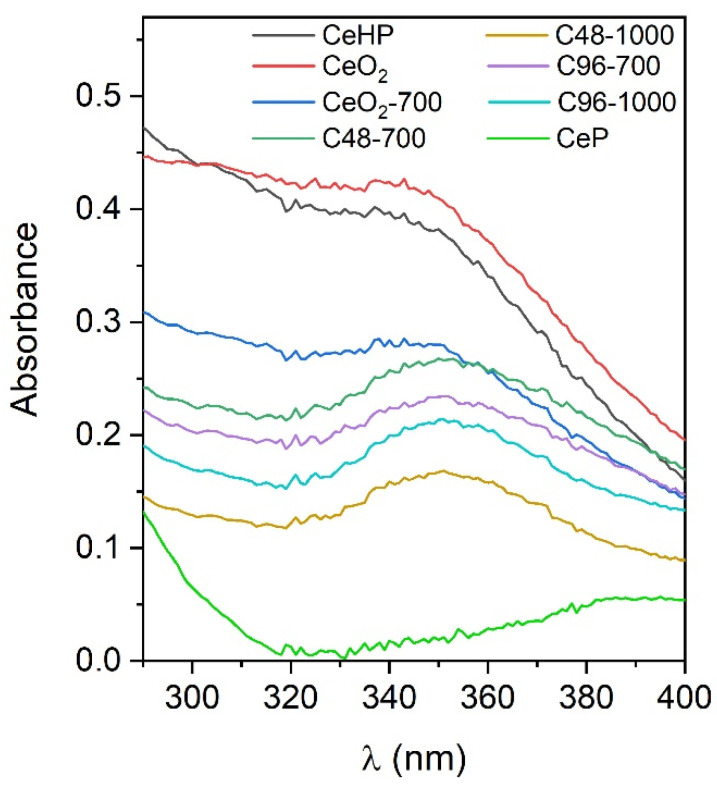
Averaged UV–vis absorption spectra of the suspensions containing ceria, cerium phosphates, and CePO_4_/CeO_2_ composites.

According to the ISO 24443-2016 international standard [23] for the correct SPF and UVAPF determination, the absorption spectra of suspensions containing UV filters should be registered a few times, just before, and just after their irradiation with UV light. Figure 8 shows the averaged UV–vis absorption spectra of suspensions containing cerium dioxide samples, CeHP, CeP, and CePO_4_/CeO_2_ composites after UV irradiation. The suspension containing CeP powder demonstrated the lowest absorption values, in the range of 290–400 nm, while CeHP and nanocrystalline CeO_2_ possessed the highest absorption. The absorption values of the suspensions containing CePO_4_/CeO_2_ composites were lower than the absorption of CeO_2_-700-containing suspension. Suspensions containing C48-700 and C96-700 composites showed a greater absorption than the corresponding suspensions containing the composites annealed at 1000 °C. The calculated values of the sun protection factor for the samples were directly dependent on the absorbance of their suspension, ranging from 2.9 for CeHP and nanocrystalline ceria (which is close to the previously published data [18,47]) to 1.0 for CeP (see Table 3). Both nanocrystalline ceria and CeHP also demonstrated the highest protection factor against UV-A radiation.

In addition, the suspensions were assessed in terms of the critical wavelength (*λ*_crit_) and UVAPF/SPF values. For all samples, these values exceeded the minimum threshold value of 370 nm and 1/3, respectively [48].

Inorganic components of sunscreen cosmetics, in addition to high SPF values, should also possess low photocatalytic activity (PCA). Thus, an assessment was made of the rate of methylene blue dye photodegradation in the presence of CeHP, CeP, CeO_2_, and C48-700 samples (the latter has one of the highest SPF values among the CePO_4_/CeO_2_ composites), in comparison with commercial titanium dioxide (Aeroxide TiO_2_ P25). 

**Figure 9:**
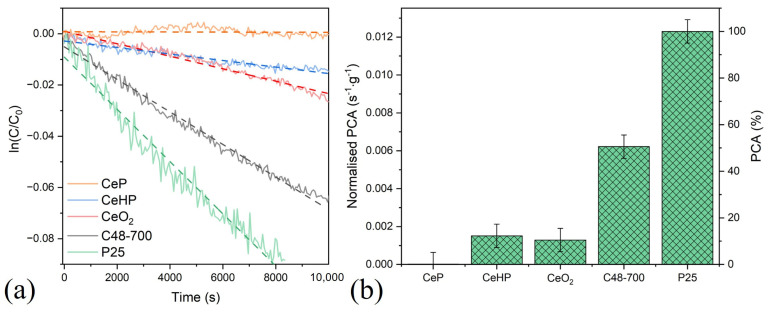
(**a**) Kinetic curves of methylene blue dye photodegradation in the presence of CeP, CeHP, C48-700, ceria, and TiO_2_ Aeroxide TiO_2_ P25 samples; and (**b**) normalised photocatalytic activity (PCA) for the corresponding samples.

Figure 9 shows that the CeP sample, having the lowest SPF value, did not possess any photocatalytic activity, which correlates well with previous reports [12,36,49]. Both ceria and CeHP samples provided almost the same rate of methylene blue photodegradation, which was approximately ten times lower than the corresponding value for TiO_2_. The low level of photocatalytic activity of nanocrystalline ceria compared with titanium dioxide is also in line with previous reports [50,51,52,53,54]. At the same time, the C48-700 composite showed a higher photocatalytic performance than CeO_2_, which can be caused by the complex interplay of several factors, such as the particle morphology, particle size, lifetime of photogenerated electron-hole pairs [55], redox switching between Ce^+3^ and Ce^+4^ in CePO_4_/CeO_2_ composites [56,57,58], etc. 

As shown previously, cerium(IV) compounds—ceria and ceric phosphate Ce(PO_4_)(HPO_4_)_0.5_(H_2_O)_0.5_—are non-toxic to mammalian cell cultures, which potentially allow them to be considered to be promising components of sunscreen cosmetics [18]. In the present study, Ce^III^PO_4_ cytotoxicity was analysed under similar conditions, in order to make the comparison of cerium(III) and (IV) phosphates more detailed.

**Figure 10:**
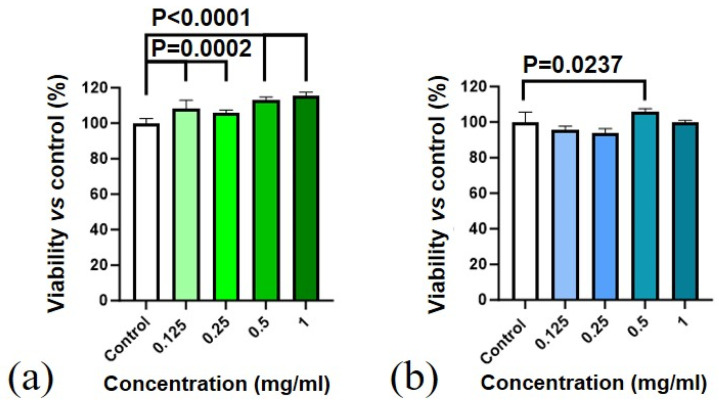
Metabolic activity of (**a**) human mesenchymal stem cells and (**b**) mouse fibroblasts of the NCTC L929 line after 48 h of cultivation with the CeP sample in concentrations of 1, 0.5, 0.25, and 0.125 mg/mL. Seeding density was 20,000 cm^−2^. M ± SD, Mann–Whitney U-test, at *p* = 0.05.

**Figure 11:**
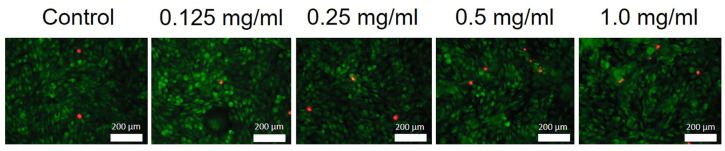
Appearance of L929 mouse fibroblasts in the presence of the CeP sample. Images were taken at 200× magnification. Scale bar—200 μm. SYTO 9 and propidium iodide dyes (live/dead test) were used. Green—live cells, red—dead cells.

The results of the MTT test for human mesenchymal stem cells and NCTC L929 mouse fibroblasts (Figure 10) showed no significant differences between the CeHP [18] and CeP samples, indicating the absence of the toxic effect in the concentration range of 0.125–1 mg/mL. The results of the live/dead assay (Figure 11) are consistent with the MTT test data. 

Although the data on the toxicity of cerium(III) ions had already been reported [59,60,61], the low toxicity of the CePO_4_ was apparently due to the extremely low solubility of monazite [62,63,64] and the absence of free cerium ions in the solution. When comparing cerium phosphates and cerium dioxide, some disadvantages of ceria should be taken into account. In particular, its pro-oxidant activity at the natural pH of the skin (~5) has been reported earlier, as well as the tendency to interact with phosphate groups of sunscreen components or cell membranes [65,66,67,68]. Biocompatibility and stability in the biological environment of the phosphate matrix make cerium phosphates the most promising candidates for use in sunscreen cosmetics; they are free from the abovementioned limitations inherent in ceria.

## 4. Conclusions

This paper presents a first comparative quantitative assessment of the UV-shielding properties (SPF, UVAPF, *λ*_crit_) of cerium(III) and (IV) phosphates, as well as Ce^III^PO_4_/Ce^IV^O_2_ composites. It has been shown that Ce(PO_4_)(HPO_4_)_0.5_(H_2_O)_0.5_ and CePO_4_ (monazite) powders, with a similar crystallite size and lamellar microstructure, have SPF values of 2.9 (like nanocrystalline ceria) and 1.0, respectively. Composites containing CeO_2_ and CePO_4_ phases have SPF values between 1.4 and 1.8, which exceed the corresponding value for the individual CePO_4_ (monazite). At the same time, CePO_4_ powder has no photocatalytic activity in the reaction of methylene blue degradation. The photocatalytic activity of Ce(PO_4_)(HPO_4_)_0.5_(H_2_O)_0.5_ is comparable with the photocatalytic activity of nanocrystalline ceria, being ten times lower than that of Aeroxide TiO_2_ P25. The Ce^III^PO_4_/Ce^IV^O_2_ composite with SPF 1.8 possesses the highest photocatalytic activity, which is only half that of Aeroxide TiO_2_ P25. The absence of cytotoxicity of CePO_4_ towards human mesenchymal stem cells and NCTC L929 mouse fibroblasts has been demonstrated by MTT and live/dead tests. Based on the whole body of data obtained, Ce(PO_4_)(HPO_4_)_0.5_(H_2_O)_0.5_ is concluded to be the most promising sunscreen component among the cerium-containing materials studied.

## Figures and Tables

**Table 1:** Synthesis parameters and designations of the cerium phosphate/ceria composites.

Sample	C48-700	C48-1000	C96-700	C96-1000
Soaking duration in 1 M NaOH solution, h	48	48	96	96
Annealing temperature, °C	700	1000	700	1000

**Table 2:** Structural parameters and crystallite sizes of ceria, cerium phosphates, and composites samples obtained by XRD analysis.

Sample	Phase Composition	Lattice Parameters	Crystallite Size, nm
CeO_2_	CeO_2_: 100 wt.%	*a* = 5.4184(7) Å	6.2 ± 0.5
CeO_2_-700	CeO_2_: 100 wt.%	*a* = 5.4121(7) Å	16.4 ± 0.5
CeHP	Ce(PO_4_)(HPO_4_)_0.5_(H_2_O)_0.5_: 100 wt.%	*a* = 21.041(1) Å *b* = 6.5644(5) Å *c* = 6.9609(5) Å *β* = 91.965(5)°	>100
CeP	CePO_4_: 96.1 ± 2.9 wt.% CeP_3_O_9_: 4.1 ± 0.3 wt.%	CePO_4_: *a* = 6,7969(2) Å *b* = 7.0202(6) Å *c* = 6.4689(5) Å *β* = 103.453(4)° CeP_3_O_9_: *a* = 11.28(1) Å *b* = 8.60(1) Å *c* = 7.345(1) Å	>100
C48-700	CeO_2_: 94.3 ± 0.6 wt.% CePO_4_: 5.7 ± 0.2 wt.%	CeO_2_: *a* = 5.4166(7) Å CePO_4_: *a* = 6.756(5) Å *b* = 6.956(5) Å *c* = 6.444(4) Å *β* = 103.83(7)°	CeO_2_: 9.7 ± 0.5 CePO_4_: 80 ± 30
C48-1000	CeO_2_: 82.5 ± 0.7 wt.% CePO_4_: 17.4 ± 0.7 wt.%	CeO_2_: *a* = 5.4116(2) Å CePO_4_: *a* = 6.793(1) Å *b* = 7.019(1) Å *c* = 6.469(1) Å *β* = 103.46(1)°	CeO_2_: >100 CePO_4_: >100
C96-700	CeO_2_: 100 wt.% CePO_4_: not detected	*a* = 5.419(1) Å	9.9 ± 0.5
C96-1000	CeO_2_: 85.6 ± 0.9 wt.% CePO_4_: 14.3 ± 1.0 wt.%	CeO_2_: *a* = 5.4125(2) Å CePO_4_: *a* = 6.786(2) Å *b* = 7.021(3) Å *c* = 6.467(2) Å *β* = 103.36(3)°	CeO_2_: >100 CePO_4_: >100

**Table 3:** Sun protection factor, UV-A protection factor, and critical wavelength values for the samples of ceria, cerium phosphates, and CePO_4_/CeO_2_ composites.

Sample	SPF	UVAPF	UVAPF/SPF	*λ*_crit_, nm
CeHP	2.9	2.5	0.9	381
CeO_2_	2.9	2.7	0.9	382
CeO_2_-700	2.0	1.9	1.0	383
C48-700	1.8	1.8	1.0	386
C96-700	1.7	1.7	1.0	385
C48-1000	1.4	1.4	1.0	385
C96-1000	1.5	1.5	1.0	386
CeP	1.0	1.0	1.0	395
TiO_2_ (anatase) [18]	2.7	2.5	0.9	
TiO_2_ (anatase) [18]	3.1	3.1	1.0	
ZnO (Z-Cote^®^) [47]	3.7	3.8	1.0	

## Data Availability

Data are contained within the article and Supplementary Materials.

## Supplementary Materials

The following supporting information can be downloaded at: https://www.mdpi.com/article/10.3390/molecules29092157/s1, Figure S1: Normalised UV-vis absorption spectra of ceria, cerium phosphates and CePO_4_/CeO_2_ composites; Table S1: EDX data for the cerium phosphate/ceria composites.
