# Supplementary material for: A Comparative Study of Cerium(III) and Cerium(IV) Phosphates for Sunscreens
Source: Molecules. 2024 May 6;29(9):2157. doi: 10.3390/molecules29092157 (PMC11085409; doi:10.3390/molecules29092157)
Supplement: Supplementary file 1 [file molecules-29-02157-s001.zip › molecules-2983025-supplementary.pdf]

Supplementary Materials

A Comparative Study of Cerium(III) and Cerium(IV) Phosphates for Sunscreens

Taisiya O. Kozlova [1], Darya N. Vasilyeva [1,2], Daniil A. Kozlov [1], Irina V. Kolesnik [1,3], Maria A. Teplonogova [1], Ilya V. Tronev [1,2], Ekaterina D. Sheichenko [1,2], Maria R. Protsenko [1,2], Danil D. Kolmanovich [4], Olga S. Ivanova [5], Alexander E. Baranchikov [1] and Vladimir K. Ivanov [1,*]

1 Kurnakov Institute of General and Inorganic Chemistry, Russian Academy of Sciences, Moscow 119991, Russia
2 Faculty of Chemistry, National Research University Higher School of Economics, Moscow 101000, Russia
3 Faculty of Materials Science, Lomonosov Moscow State University, Moscow 119991, Russia
4 Institute of Theoretical and Experimental Biophysics, Russian Academy of Sciences, Pushchino 142290, Russia
5 Frumkin Institute of Physical Chemistry and Electrochemistry, Russian Academy of Sciences, Moscow 119071, Russia
* Correspondence: van@igic.ras.ru

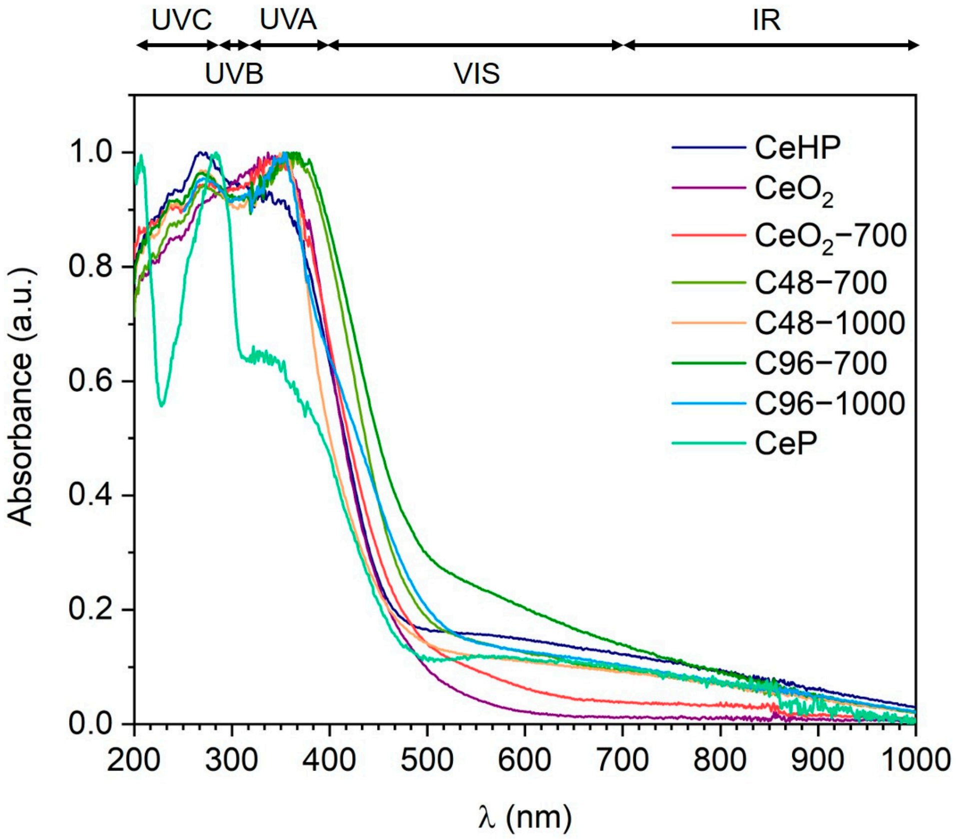

Figure S1. Normalised UV-vis absorption spectra of ceria, cerium phosphates and $CePO_4/CeO_2$ composites.

Table S1. EDX data for the cerium phosphate/ceria composites.

Sample	C48-700	C48-1000	C96-700	C96-1000
Ce:P ratio	7.8±2.6	8.6±2.6	7.0±1.0	6.1±1.2
